# Functional Method of Designing Self-Compacting Concrete

**DOI:** 10.3390/ma14020267

**Published:** 2021-01-07

**Authors:** Tomasz Rudnicki

**Affiliations:** Faculty of Civil Engineering and Geodesy, Military University of Technology in Warsaw, 2 Gen. S. Kaliskiego St., 01-476 Warsaw, Poland; tomasz.rudnicki@wat.edu.pl

**Keywords:** SCC, designing SCC, plastic viscosity, compressive strength, concrete mix flow

## Abstract

The article presents a new functional method of designing self-compacting concrete (SCC). The assumptions of the functional method of designing self-compacting concrete were based on the double coating assumption (i.e., it was assumed that the grains of coarse aggregate were coated with a layer of cement mortar, whereas the grains of sand with cement paste). The proposed method is composed of four stages, each of which is responsible for the selection of a different component of the concrete mix. The proposed designing procedure takes into consideration such a selection of the mineral skeleton in terms of the volumetric saturation of the mineral skeleton, which prevents the blocking of aggregate grains, and the designed liquid phase demonstrated high structural viscosity and low yield stress. The performed experimental studies, the simulation of the elaborated mathematical model fully allowed for the verification of the theoretical assumptions that are the basis for the development of the method of designing self-compacting concrete.

## 1. Introduction

High architectonic and endurance requirements that are currently placed before the constructed engineering facilities force the constant progress and development of the technology of the basic construction material, which is (and will remain for a long time) cement concrete.

Currently, an element that has become a big technological impediment is the necessity of ensuring the required compacting of cement concrete in reinforced concrete buildings, demonstrating very dense construction reinforcement. This refers above all to the construction of large sized facilities with a dense system of construction reinforcement, which may cause a lack of homogeneity of the structure, resulting from the insufficient vibration of the fresh concrete mix. The expected durability and ensuring the appropriately smooth external structure of the buildings have caused the dynamic development of concretes demonstrating high liquidity, which do not require the process of mechanical compacting.

The effect of a range of design works was the creation (in Japan, Sweden, France, and in Germany) of a SCC concrete composite called self-compacting concrete, which, thanks to its specific properties, easily fills the formwork, self-compacts, and deaerates under the impact of its own weight. Its high liquidity results in the fact that it reaches locations that are hard to access (densely reinforced ones) and surrounds the construction reinforcement very well. Thanks to the increased viscosity of the cement paste, all the components of this concrete flow simultaneously, suspended in a dense stable structure without causing segregation and sedimentation. This is possible thanks to the increase in the share of the dust fraction below 0.125 mm and the application of a new generation of superplasticizers that increase the viscosity of make-up water and limit the phenomenon of segregation of coarse and very fine components of the concrete mix mineral skeleton.

Since the elaboration of this concrete by Okamura and Ozawa [1,2,3], numerous works have allowed for a greater familiarity with the properties of this concrete and, above all, of the concrete mix, which ensures its specific characteristics and has enabled elaborating standards for analyzing them [4,5,6,7,8,9,10,11,12,13,14,15,16,17,18,19,20,21,22,23,24,25,26,27,28,29,30,31,32,33,34]. This required a relatively long time, if we take into consideration the first applications of this concrete, for example, in the construction of the “Trans Tokio Bay Bridge” constructed across Tokyo in 1988 because the series of EN 12350 [35] was introduced in 2010. The first method of designing self-compacting concrete was elaborated by Okamura and Ozawa [1,2,3] and has been used up until today by numerous authors [36,37,38,39,40,41,42,43,44,45,46,47,48,49,50,51]. The “flow” of the concrete mix depends, above all, on the content of the coarse aggregate, which decides on the friction force in reference to the remaining components of the mix, increasing the flow resistance and reducing its speed. Due to this, the self-compacting mix has a lower content of coarse aggregate, and the aggregate also has a smaller diameter, not exceeding 16 mm, and frequently even 12 mm. The mix includes more mortar and has a much higher sand content. For this purpose, Okamura and Ozawa [20,21,22,52] introduced a water/dust coefficient equal to 1. The mass relationship of coarse aggregate to fine aggregate and to the cement matrix should be the following percentage: 50:20:30 [30]. De Larrard [8,9,15,34] developed a scientific method of designing the composition of the concrete mix that he published together with Sedran [15,34] in 1994, and in Poland, he discussed it during the “Dni Betonu” (“Concrete Days”) Conference in 2004 [9]. A method worth mentioning is that of Petersson et al. [24,53], in which the amount of paste is selected using its rheological properties. A method which is also interesting is that of Farraris et al. [9] as well as the German method by Grube and Rickert [54], and that by Billberg [5,6,55] and by Van and Montgomery [20,37]. Grube and Rickert [54] indicated that the share of paste they recommended should be 40%, whereas the share of sand should be 25% and the share of coarse aggregate 35%. It seems that despite taking into consideration various kinds of mineral additives including silica fumes, the method of Serdan and de Larrard [8,9,15,34] is used relatively rarely. The method by Okamura and Ozawa [1,2,3] is still most popular. Moreover, Spanka et al. [56] have more broadly considered the share of the dust fraction in designing, aside the cement, in the self-compacting concrete mix. Venkateswara Rao et al. [40] suggested a very simple method of designing concrete mix and verified it experimentally; until now, it has not been applied by other authors. There is a range of works devoted to the topic of designing self-compacting concrete mixes [1,2,3,4,5,6,7,8,9,10,11,12,14,15,16,17,20,21,22,23,24,55,56,57,58]. Research works [4,5,6,7,8,9,10,59,60,61,62,63,64] conducted in the field of the development and application of this kind of composite have been carried out due to economic reasons (shortening of the construction time) [36] as well as due to ecological matters (a lack of the burdensome noise of the operating vibrators compacting the concrete) and technical benefits arising from the resistance to the effect of the corrosion environment and of the thawing agents in the form of salts applied during the glaze. A very important advantage of SCC is high compressive strength resulting from the lowered water–cement ratio (w/c) as well as from a tightly designed aggregate skeleton.

Initially, the elements considered the basic properties of a self-compacting concrete mix were the flow and the deaeration as well as compacting under the influence of its own mass. The above-mentioned properties did not characterize SCC in a full way, therefore observations of additional properties began such as flow through dense reinforcement, the dynamics of the flow, tightness and the ease of filling in forms, maintaining consistency in time, and the surface aesthetics. The development of a new technological field, which self-compacting concrete undoubtedly is, has resulted in the creation of a range of design works, the aim of which was obtaining concrete that meets the high technological and utilization requirements with very high liquidity.

In Europe, one of the first applications of SCC was conducted in Sweden [7] where in 1993, intense studies and laboratory trials were begun, with special emphasis on applying this concrete in road construction engineering. As it later turned out, the basic difference in comparison to traditional concrete refers to a change of the composition, namely the introduction of additional amounts of dusty parts <0.25 mm in the form of fillers and a chemical admixture of a new generation of a very strong liquidating and stabilizing effect. Currently, the construction industry faces high challenges and changes, i.a., referring to the increase in the durability and more effective construction in terms of both, the technology as well as the reduction in the possible costs [34]. Such clearly set goals have resulted in the emergence of the concept of the development of the technology of concretes of very high strength and liquidity, which, by their assumption, eliminate the compacting work performed through mechanical vibrating.

The aim of the work was to propose a design procedure that takes into consideration such a selection of the mineral skeleton in terms of the volumetric saturation of the mineral skeleton, which prevents the blocking of aggregate grains, and the designed liquid phase demonstrated high structural viscosity and low yield stress.

## 2. Materials and Methods

### 2.1. Materials

The research work included the use of:

- two types of cement complying with PN-EN 197-1 [65], the cement CEM I 42.5 R (Lafarge, Bielawa, Poland) and CEM II B-S 42.5 N (Lafarge, Bielawa, Poland);
- in order to increase the viscosity of the concrete mix and reduce the amount of cement, a mineral additive was used in the form of limestone dust complying with the PN-EN 13043 [66] with the following density of 2.77 g/cm^3^;
- two types of aggregate were used in the studies, natural aggregate-gravel-fraction 2/8 and 8/16 mm of a density of 2.65 g/cm^3^ and water absorption of 1.60%, and crushed-stone aggregate-basalt 2/8 and 8/16 with a density of 2.95 g/cm^3^ and water absorption of 0.50%, and natural sand-fraction 0/2 with a density of 2.64 g/cm^3^;
- A third-generation high-range/strong water-reducing admixture CP and VMA viscosity regulators complying with PN EN 934-1 [67] and PN-EN 934-2 [68] (a modified polycarboxylic high-range water-reducing admixture in liquid form with a density of 1.02 to 1.07 g/cm^3^);
- tap water complying with PN-EN.

### 2.2. Functional Design Procedure

While performing an analysis of the mechanism of operation of the self-compacting of concrete, one can assume—in accordance with the assumptions of the physicochemical mechanics of dispersion systems—that it is a dispersion system composed of the dispersed phase (mineral skeleton above 0.25 mm) and of the liquid phase (water, cement, filler, superplasticizer, and fine sand <0.25 mm) [10,18,36]. The assumptions of the functional method of designing self-compacting concrete were based on the method by Professor Paszkowski, the so-called double coating method (i.e., it was assumed that the grains of coarse aggregate were coated with a layer of cement mortar, whereas the grains of sand with cement paste). It was assumed that the functional method is composed of four stages, each of which is responsible for the selection of a different component, and the final verification of the properties takes place at the stage of the trial batch. The stages of the functional method are presented in Table 1:

### 2.3. Designing the Cement Paste

**Figure 1:**
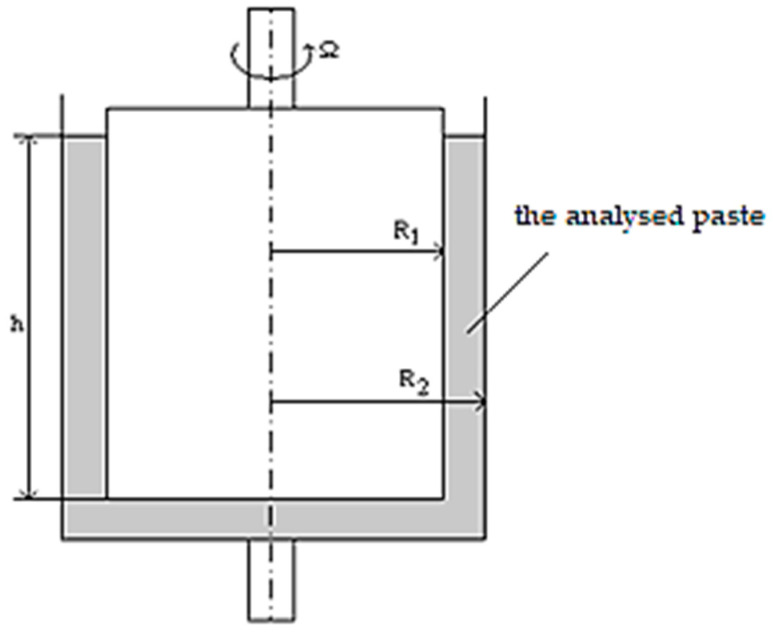
A schematic image of the Rheotest 2 viscometer with coaxial cylinders, R_1_—inner diameter, R_2_—outer diameter, Ω—revolutions of the Rheotest 1/min, h—sample height.

The first stage of design was based on designing a cement paste that would demonstrate appropriate viscosity, viscosity hysteresis loop, and flow greater than 18 cm measured using the Southard viscometer. The first part of the laboratory experiment was based on rheological studies performed with the use of a rotary viscometer with coaxial cylinders, Rheotest 2, for the designed pastes with various water–cement ratios w/c (0.6; 0.55; 0.50; 0.45; 0.40) for two different cements (CEM I and CEM II), which allow for the assessment of the behavior of these pastes with a decreasing amount of water and a constant amount of cement. This device was used for determining the viscosity of the paste and the flow curves in the shear stress-shear rate system. The flow curves describing the mechanism of behavior of the continuous phase by creating the hysteresis loop were formed as a result of the increase and decrease of the shear rate. Additionally, examinations with a Southard viscometer were performed in order to determine the standard flow of the paste. Subsequently, in a further part of the experiment, the paste with the determined water–cement ratio (w/c) was subjected to modification performed using very strongly liquidating superplasticizers in the following doses of 0.5, 1.0, 1.5, and 2.0% in reference to the mass of the cement. The scheme of analyzing the pastes in the Rheotest 2 is presented in Figure 1:

The values of the shear stress *τ* and of the shear rate gradient *γ* are associated with the radius of the coaxial cylinder system. Shear stress is expressed using Formula (1):

(1) τ=z·α

where: τ is the shear stress, dyn/cm^2^, Pa^−1^; z is the cylinder constant, dyn/cm^2^; and *α* is the value read from the indicating instrument.

For the determined values of shear stress τ and for the read value of the shear rate gradient γ, it is possible to calculate dynamic viscosity η:

(2) $$\eta =\frac{\tau }{\gamma }$$

where η is the dynamic viscosity; τ is the shear stress, dyn/cm^2^, Pa^−1^; and γ is the shear rate gradient, s^−1^.

The next step of stage one is to calculate the minimum amount of the cement paste $Z_{c\;min}$, allowing for the flow of concrete on the basis of the determined free space in the mineral mix and the specific surface area of the aggregate skeleton according to Formula (3):

(3) $$Z_{c\;min}=Z_{1}+Z_{2}$$

**Figure 2:**
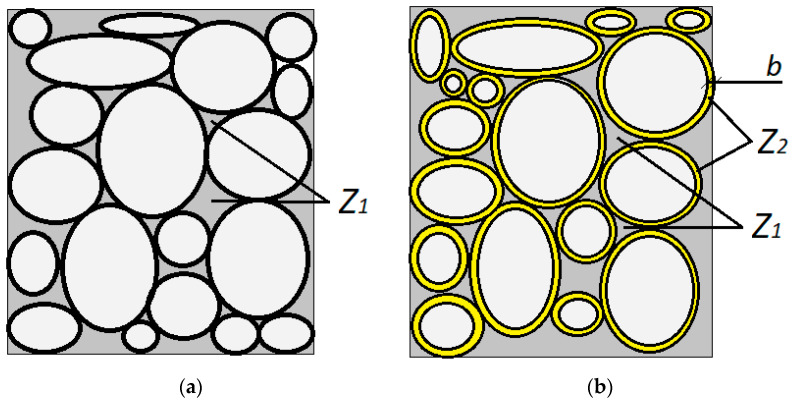
The change in the structure of the aggregate skeleton as a result of coating the grains of the coarse aggregate with paste (the liquid phase). (**a**) normal concrete; (**b**) SCC.

The graphical representation of the amount of paste described with Formula (3) is presented in Figure 2:

The minimum amount of paste filling the free space between the grains of aggregate $Z_{1}$ is calculated using Formula (4) and the minimum amount of paste that causes the separation of the aggregate grains to the distance *b* is calculated according to Formula (5) and is marked as $Z_{2}$:

(4) $$Z_{1}=\frac{P_{min}\cdot \rho_{wz}}{\rho_{o\;max}}$$

where $Z_{1}$ is the minimum amount of paste filling the free space in the mineral mix, g_z_/g_k_; $P_{min}$ is the experimentally determined minimum free space in the mix, %; $\rho_{wz}$ is the density of the cement paste, g/cm^3^; and $\rho_{0\;max}$ is the maximum bulk density, g/cm^3^.

(5) $$Z_{2}=P_{w}\cdot b\cdot \rho_{wz}$$

where $Z_{2}$ is the minimum amount of paste that causes the overfilling of free spaces in the mineral mix, g_z_/g_k_; $P_{w}$ is the specific surface area of the aggregate, cm^2^/g; and b is the thickness of the membrane on the aggregate, cm.

Finally, the demand for the minimum amount of cement paste allowing for the flow of concrete is determined according to Formula (6):

(6) $$Z_{c\;min}=\left(\frac{P_{min}}{\rho_{o\;max}}\cdot \;\rho_{wz}+P_{w}\cdot b\cdot \rho_{wz}\right)$$

According to the assumptions of this method, the determined minimum amount of the cement paste $Z_{c\;min}$ should allow for obtaining the liquidity of the designed concrete. The final amount of paste (the liquid phase) was determined using Formula (7):

(7) $$Z=Z_{c\;min}\cdot K$$

where Z is the final amount of the paste in the concrete mix, kg/m^3^; and K is the amount of fine and coarse aggregate, kg/m^3^.

According to the formula for absolute volume:

(8) $$\frac{Z}{\rho_{wz}}+\frac{K}{\rho_{k}}=1000$$

where $\rho_{k}$ is the density of the mineral aggregate skeleton, g/cm^3^.

The final amount of aggregate [69,70,71,72,73] in the concrete mix is calculated using Formula (9):

(9) $$K=\frac{1000}{\frac{Z_{c\;min}\;}{\rho_{wz}}+\frac{1}{\rho_{k}}}$$

In order to verify the correctness of the calculations of all the components of the concrete mix, it is necessary to use the formula for the so-called absolute volume (or the tightness condition), which has the following form:

(10) $$\frac{c}{\rho_{c}}+\frac{k}{\rho_{k}}+\frac{p}{\rho_{p}}+\frac{d}{\rho_{d}}+\frac{w}{\rho_{w}}+\frac{m}{\rho_{m}}=1000$$

where c is the amount of cement, kg/m^3^; k is the amount of coarse aggregate, kg/m^3^; p is the amount of sand, kg/m^3^; d is the amount of the chemical admixture (the superplasticizer), kg/m^3^; w is the amount of water, kg/m^3^; m is the amount of limestone dust, kg/m^3^; $\rho_{c}$ is the density of the cement, kg/dm^3^; $\rho_{k}$ is the density of the aggregate, kg/dm^3^; $\rho_{p}$ is the density of the sand, kg/dm^3^; $\rho_{d}$ is the density of the chemical admixture (superplasticizer), kg/dm^3^; $\rho_{w}$ is the density of water, kg/dm^3^; and $\rho_{m}$ is the density of the limestone dust, kg/dm^3^.

### 2.4. The Selection of the Grading of the Mineral Mix with the Use of the Maximum Bulk Density Method

The second stage of the functional design of the self-compacting concrete mix is the selection of the grading of the mineral mix by determining the tightness of the aggregate skeleton and the maximum bulk density. This stage consisted of the optimum selection of the particular fractions of aggregate in such a way as to obtain the maximum degree of saturation (i.e., minimum vacuum). The methodology for performing this was based on the formerly [66] applied method of selecting the grading for designing mastic asphalt. Minimum vacuum in the mineral skeleton was obtained by gradually adding finer fractions of aggregate. The free spaces formed in the coarse aggregate should be filled with finer grains and sand and the free spaces formed in the sand are filled with the use of a mineral additive in the form of (e.g., limestone dust) [62]. At this stage, the specific bulk density for consecutively designed mineral mixes was determined, starting with the volumetric selection from the biggest fraction of aggregate and finishing with the inert filler. In order to determine the maximum bulk density, Formula (11) was applied:

(11) $$\rho_{0}=\frac{m}{V_{k}}$$

where m is the mass of the analyzed aggregate in the cylinder, g; and $V_{k}$ is the volume of the aggregate in the cylinder, cm^3^.

Determining the maximum density of the mineral skeleton should start from the coarsest fraction of the rock material (i.e., from the fraction of 8/16 mm gravel and small but even portions of material with smaller grading—2/8 mm—should be added to it). This activity should be repeated until the moment when the maximum bulk density is obtained and the subsequent attempt to add 2/8 material causes a decrease in the density of the mix. The mix of 8/16 and 2/8 gravel for which maximum bulk density is obtained is the desired starting material for subsequent attempts; the difference is that now finer materials are added (i.e., sand of 0/2 mm grading).

Finer material should be subsequently added in this way until, in the final part of this study, the mineral filler in the form of inert limestone dust is added and the mineral mix obtained in this experiment demonstrates maximum tightness and a minimum amount of free spaces. The effect of this part of the design is obtaining a mineral mix of maximum tightness resulting from appropriate proportions of particular mineral components. Subsequently, the specific density (without air voids) of the mineral components should be determined using the Le Chatelier Flask. Specific density should be calculated using Formula (12):

(12) $$\rho_{w}=\frac{m}{V}$$

where m is the mass of a dry, powdered specimen of the material, g; and V is the volume of the powdered specimen of the aggregate placed in the flask, cm^3^.

On the basis of the experimentally determined maximum bulk density of the aggregate skeleton and the density of the mineral mix determined using the Le Chatelier Flask, it is possible to determine the amount of free spaces in the designed mineral mix. The free space in the designed mineral mix is calculated according to Formula (13):

(13) $$P_{min}=1-\frac{\rho_{0\;max}}{\rho_{w}}$$

where $P_{min}$ is the experimentally determined minimum free space in the mix, %; $\rho_{w}$ is the density of the mix, g/cm^3^; and $\rho_{0\;max}$ is the maximum bulk density, g/cm^3^.

### 2.5. Determining the Grading and the Specific Surface Area (External) of the Aggregate Skeleton (Stage III)

Stage III of design is determining the grading and, on its basis, calculating the specific surface area (external) of the designed aggregate skeleton. This stage is based on the assumption [41] that the paste should cover all the grains of the aggregate with a layer of paste of the thickness b, resulting in the effect of overfilling with paste. This stage aims at creating a non-contact structure because the aggregate skeleton, tightly designed in stage I, causes an increase in internal friction between the grains of the aggregate, which precludes the free flow of the concrete mix.

The reason for the occurrence of the blockade is the too close contact between the most coarse grains, resulting in the drastic increase of the yield stress, which hinders the flow and causes the blocking of the mineral skeleton [12].

The aggregate skeleton designed this way should demonstrate the smallest possible specific surface area, which may be calculated according to Formula (14):

(14) $$P_{w}=\frac{\sum f_{i}\cdot P_{i}}{100}$$

where $P_{w}$ is the total specific surface area of the aggregate, cm^2^/g, $f_{i}$ is the content of particular fractions, %; and $P_{i}$ is the specific surface area of a particular fraction, cm^2^/g.

The specific surface area constitutes the area of the unfolded surface of all the grains of aggregate included in a given unit of volume. The higher the value of the specific surface area, the higher the demand for the content of the liquid phase. Knowing the grading and the designed grain size distribution curve, on the basis of Table 2, it is possible to calculate the specific surface area [29] of the pebble aggregate with the following density of 2.65 kg/dm^3^.

On the basis of the calculated specific surface area and the thickness of the *b* membrane coating the aggregate grains, it is possible to, with high accuracy, calculate the minimum amount of cement paste necessary for ensuring the proper flow of the self-compacting concrete mix.

### 2.6. Creating Trial Batches of the Designed Concrete Mixes (Stage IV)

The fourth stage of designing self-compacting concrete is based on the verification of the parameters of the concrete mix and of the hardened concrete through the creation of trial batches. Determining the parameters obtained by the self-compacting concrete mix may take place through a 4-grade assessment based on the L-box, the V-funnel, and the Abrams inverted cone. This stage of analysis is relatively simple, but very important because only here is it possible to observe the actual behavior of the mix and introduce necessary corrections to its composition.

Additionally, for particular batches, the properties of hardened concrete were determined such as compressive strength after seven, 28, and 90 days, water absorption, resistance to the effect of freeze, and the density.

## 3. Results

This method was used to design the grading of the mineral skeleton of mastic asphalt, because it allowed for the design of a tight aggregate skeleton in an easy and accurate way, based on the mineral materials applied in the local construction market.

At this stage of design, the main aim is to design a cement paste that would demonstrate appropriate viscosity, viscosity hysteresis loop, and flow greater than 18 cm as determined using the Southard viscometer.

### 3.1. The Selection of the Type of Cement and Determining the Water–Cement Ratio (w/c)

In this part, the research experiment was based on rheological analyses (performed with the use of the Rheotest 2 rotary viscometer and the Southard viscometer) of pastes demonstrating various water–cement ratios (w/c), starting from 0.6, 0.55, 0.50, 0.45, and ending with 0.40. All of the analyzed pastes were made of two cements (CEM I 32.5 R and CEM II/B-S 32.5 manufacturer—Lafarge).

The measured values of the paste viscosity and the flow curves that formed as a result of the increase and decrease of the shear rate are demonstrated in the graph presenting the flow curves for the paste on the basis of CEM II with the water–cement ratio (w/c) = 0.5.

**Figure 3:**
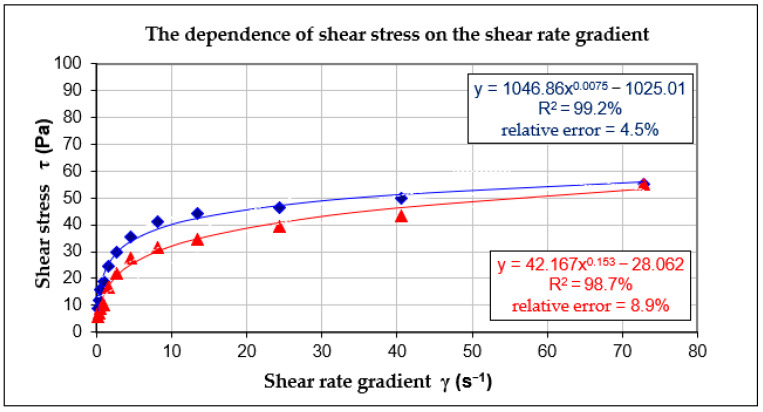
The dependence of shear stress on the shear rate of the cement paste created on the basis of CEM II with the water–cement ratio (w/c) = 0.50.

The mathematical nature of the flow curves in Figure 3 is presented using quasi-Newton nonlinear estimation exponential curves. In order to calculate the surface area of the viscosity hysteresis loop, the integral calculus was applied in the measurement borders of the shear rate gradient.

In this way, the following was obtained:
Curve 1 (upper):$Y=1046.86\cdot x^{0.0075}-1025.01$Curve 2 (lower):$Y=42.167\cdot x^{0.153}-28.062$

In the integral function, the expression had the following form:

$$\int_{0}^{72.9}\left(1046.86\cdot x^{0.0075}-1025.01\right)dx-\int_{0}^{72.9}\left(42.167\cdot x^{0.153}-28.062\right)dx=407.887$$

**Figure 4:**
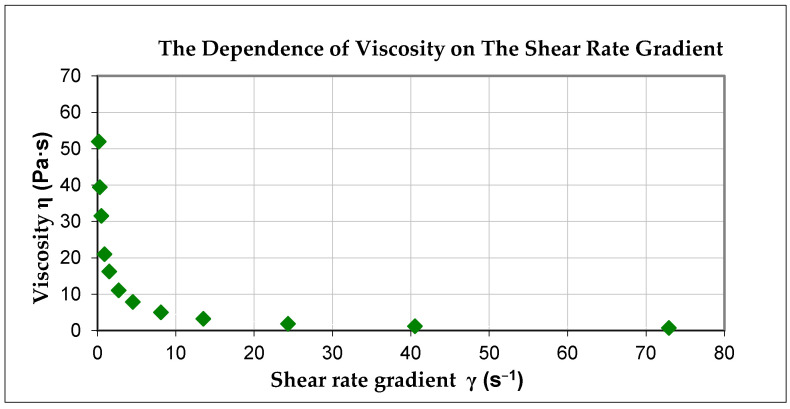
The dependence of viscosity on the shear rate gradient for the cement paste based on CEM II and the water–cement ratio (w/c) = 0.50.

In accordance with the principles of the integral calculus of the polynomial function in determined limits of integration, the surface area of the viscosity hysteresis loop obtained for the given set was 407.887. Figure 4 presents the graph of the dependence of viscosity on the shear rate gradient for the cement paste based on CEM II/B-S and the water–cement ratio (w/c) = 0.5.

The results of the performed studies for two types of cement and for various water–cement ratios (w/c) in the scope of the dependence of the viscosity of the cement paste on the type of cement and the water–cement ratio (w/c) are presented in Table 3.

Moreover, in order to verify Stage I, analysis of the pastes was performed using the Southard viscometer in which the flow of the paste was measured in cm and is presented in Table 4.

On the basis of the conducted research, the paste from the CEM II/B-S cement with the water–cement ratio (w/c) = 0.5 was selected.

### 3.2. The Selection of the Type and the Amount of the Superplasticizer

Next, for the selected water–cement ratio (w/c) = 0.5 and the CEM II/B-S 32.5 cement, the second part of the experiment was started, which consisted in the modification of the selected suspension with a third-generation high-range/strong water-reducing admixture CP and VMA viscosity regulators complying—CP1, CP2, CP3—in the following doses of 0.5, 1.0, 1.5, and 2.0% in reference to the mass of the cement, checking the compatibility of the admixture with the cement. The obtained study results related to the viscosity area of the hysteresis loop are presented in Table 5 and Table 6.

During the determining of both, the viscosity of the paste as well as of the viscosity area of the hysteresis loop, the best results were achieved for the a modified polycarboxylic high-range water-reducing admixture CP1 in the amount of 1.0% in reference to the mass of the cement. For the purpose of confirmation, the standard consistency test was performed using the Southard viscometer according to PN-86/B-04360 [74].

The obtained results confirmed the rightness of both the selection of the CP1 superplasticizer as well as of the liquidating dose in the amount of 1.0%.

### 3.3. The Selection of the Grading of the Mineral Mix

The experimental verification of the theoretical assumptions of the selection of the dispersed phase was begun from the selection of the grading of the mineral mix by determining the tightness of the aggregate skeleton and determining the maximum bulk density according to the mastic asphalt design method adopted for this purpose. For this purpose, the maximum bulk density and the minimum amount of free spaces were determined for two different types of aggregate (8/16 and 2/8 mm gravel and 8/16 and 2/8 mm crushed basalt) and for 0/2 mm natural sand as well as for a mineral filler.

**Figure 5:**
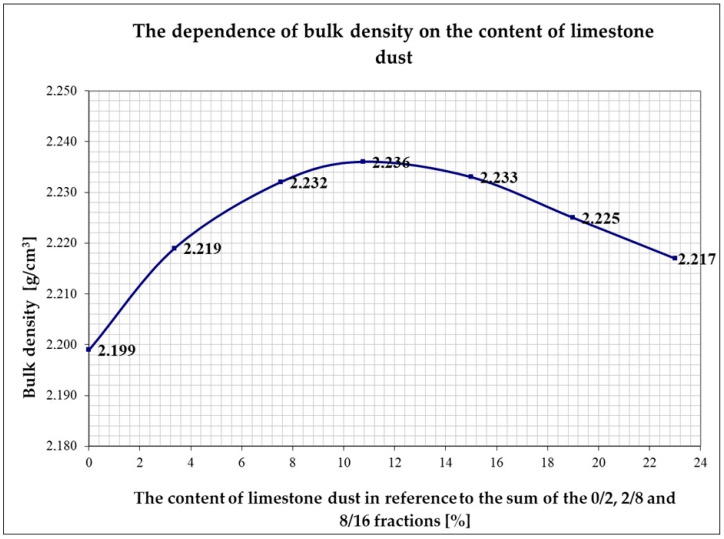
The dependence of the bulk density of the mix of 8/16 mm and 2/8 mm gravel and 0/2 mm sand in reference to the filler in the form of limestone dust.

For this purpose, the action started from the 8/16 fraction in a constant amount of 250 g and consecutively, finer 2/8 gravel was added in equal amounts of 30 g until obtaining the maximum density of the mix. These activities were repeated 10 times in order for the achieved result to be statistically reliable. The maximum bulk density equal 1.682 g/cm^3^ of the mineral mix was obtained for 73% of 2/8 gravel in reference to the 8/16 fraction. Finer material was added to the constant amount of 8/16 mm (200 g) and 2/8 mm (146 g) gravel determined in this way in order to fill the free spaces in the mineral mix. This material was 0/2 mm sand in equal portions, per 30 g. The maximum bulk density equal to 2.208 g/cm^3^ of the mineral mix was obtained for 61% of sand in reference to the sum of the 8/16 and 2/8 fraction. The finest material in the form of the limestone filler in the amount of per 15 g was added to the constant amount of 8/16 mm (150 g) and 2/8 mm (109.5 g) gravel and of 0/2 mm sand (158 g) determined in this way. The tabular and graphical juxtapositions of the actions are presented below in Table 7 and Figure 5.

The maximum bulk density equal of 2.236 g/cm^3^ of the mineral mix was obtained for 10.77% of limestone dust in reference to the sum of the fractions of 8/16 mm and 2/8 mm gravel and 0/2 mm sand.

### 3.4. Stage III—Determining Free Spaces in the Mineral Mix

On the basis of the conducted studies related to determining the maximum bulk density, compositions of mineral mixes based on natural and crushed-stone aggregate were obtained. These compositions demonstrate the minimum amount of free spaces that have been juxtaposed in Table 8.

Subsequently, the Le Chatelier Flask was used to experimentally determine the specific density (without air voids) of the used materials, and the results are presented in Table 9:

On the basis of the determined maximum density of the mineral mix and of the density of particular materials determined using the Le Chatelier Flask, the amount of free spaces in the designed mineral mix was calculated according to the formula:

$$P_{\min żwir}=\left(1-\frac{2.2382}{2.6475}\right)\times 100\%=15.46\%$$

The minimum amount of free spaces determined experimentally for the crushed-stone aggregate (basalt) has the following value:

$$P_{\min bazalt}=\left(1-\frac{2.420}{2.8268}\right)\times 100\%=14.39\%$$

### 3.5. Determining the Grading and the Specific Surface Area Experimentally for the Natural Aggregate Mix

Such a tight structure of the mineral skeleton, obtained during designing Stage I cannot guarantee the flow of the concrete mix. Therefore, out of the designed tight aggregate skeleton, it is necessary to create a mineral skeleton of a dispersive structure allowing for free flow of the self-compacting concrete mix.

At Stage III of the research experiment, the grading (size distribution) of particular components was determined and is included in Table 10.

Calculating the specific surface area (external) of the aggregate skeleton in order to separate the tightly positioned grains of the aggregate to the distance of the assumed membrane of the thickness “b” was performed on the basis of the designed compositions of mineral mixes for natural and crushed-stone aggregates (Table 11).

The minimum amount of the *Z*_2_ paste is the product of the specific surface area *P_w_*, the coating radius *b* (equal: 50 μm for aggregate >2 mm and 6 μm for aggregate <2 mm) and the density of cement paste $\rho_{wz\;}$= 1.81 g/cm^3^ with a determined water–cement ratio (w/c) = 0.5.

For a mineral skeleton designed this way, the minimum amount of paste for gravels is *Z*_2_ = 0.1217 g_z_/g_k,_ and for basalt aggregates *Z*_2_ = 0.1076 g_z_/g_k_.

### 3.6. Calculating the Minimum Amount of the Cement Paste

The third stage of design ends with determining the minimum amount of cement paste according to Formula (5). For gravel aggregate and paste with the water–cement ratio (w/c) = 0.5 with the dosage of 1.0% of CP1 in reference to the cement mass, it is:

$$Z_{c\min żwir}=\left(\frac{0.1546}{2.2382}\times 1.81+0.1217\right)=0.2467\;g_{z}/g_{k}.$$

The final amount of gravel aggregate in the concrete mix calculated using Formula (9) for the gravel aggregate is:

$$K=\frac{1000}{\frac{0.2467}{1.81}+\frac{1}{2.6475}}=1946\;\mathrm{kg}/m^{3}.$$

In accordance with earlier assumptions, the final amount of cement paste (the liquid phase) according to Formula (6) is:

$$Z=0.2467\times 1946=480\;\mathrm{kg}/m^{3}$$

The minimum amount of cement paste according to Formula (5) for basalt aggregate and paste with the water–cement ratio (w/c) = 0.5, with the dosage of 1.0% of CP1 in reference to the cement mass is:

$$Z_{c\min bazalt}=\left(\frac{0.1439}{2.420}\times 1.81+0.1079\right)=0.2155\;g_{z}/g_{k}.$$

The final amount of basalt aggregate in the concrete mix calculated using Formula (9) for gravel aggregate is:

$$K=\frac{1000}{\frac{0.2155}{1.81}+\frac{1}{2.8268}}=2109\;\mathrm{kg}/m^{3}.$$

In accordance with earlier assumptions, the final amount of cement paste (the liquid phase) according to Formula (6) is:

$$Z=0.2155\times 2109=454.5\;\mathrm{kg}/m^{3}.$$

The final stage of design according to the new volumetric method was juxtaposing the recipes for the concrete mix, which is supposed to meet the features of self-compacting in Table 12:

At this stage of design, the decision was made that full analyses verifying the rheological and mechanical characteristics were to be conducted for all recipe compositions that differed from one another in terms of the amount of the filler. This aimed at verifying the decision made during design, which referred to the amount of the applied filler and the influence of the amount of the filler on the self-compacting properties of the concrete mixes.

The juxtaposition of the compositions of all the recipes that were subjected to full verification of the rheological characteristics during analyses performed on concrete mixes in fresh and hardened condition is presented in Table 13.

Recipe numbers SCC 4-G and SCC 11-B were designed using the new method. The remaining ones were rejected during subsequent stages of design.

### 3.7. Mathematical Modeling of the Designing Method

Modeling phenomena and processes that occur in nature, with the use of a specialized mathematical and statistical apparatus, comes down to several significant elements [54,75,76]. The most important of them is to conduct an empirical study that results in obtaining the primary statistical material. The construction of the model of the formation of a dependent variable, set against the background of an independent variable or variables, is based on finding an adequate analytical form of the functional relationship occurring between these variables [22]. The estimation of the structural parameters of the model on the basis of statistical material obtained in an empirical study with the use of estimation methods, can be used for the verification of the obtained model. In the case of modeling the process of selection of the mineral skeleton of the self-compacting concrete, the research problem was based on the identification of the dependence between the density of the mineral mix and its total mass during the determination of the minimum free space between the grains. This, in turn, with the help of the obtained model expressed by Equation (15), allows for determining the structure of the mineral skeleton according to the criterion of maximum density for every mix of components.

The matter which becomes crucial is the selection of an appropriate analytical form of the model in conditions of nonlinearity of the connection between the density and the total mass of the mineral mix [77]. Nonlinear models may be divided into those which—with the help of appropriate transformations of the explained variable, of the explanatory variables, of the structural parameters and of the disturbance term—may be transformed into a linear form, and those for which there is no transformation, changing a given model to a linear form. The first ones are referred to as improper nonlinear models, the second ones are *sensu stricto* nonlinear models, referred to as proper nonlinear models. While in the case of models that may undergo linearization, it is possible to use the least squares method estimators, however, in the process of the estimation of proper nonlinear models, this is not always possible. In such situations, methods of estimation of structural parameters of nonlinear models are applied, which are based on iterative algorithms that minimize the loss function proposed by A. Wald in 1939. The loss function defined as the sum of the squares of deviations of the observed values in reference to the theoretical values is most frequently applied and can be recorded in the following way:

(15) $$L=\sum_{i=1}^{n}{\left(y_{jt}-{\hat{y}}_{jt}\right)}^{2}$$

where $y_{jt}$ is the observed values of the explained variable (density); ${\hat{y}}_{jt}$ is the theoretical values of the explained variable (density); *n* is the number of observations.

If the loss function (15) achieves the minimum in the estimation process, then the estimators may be referred to as least squares estimators. The most frequently applied nonlinear estimation algorithms with dedicated computer software are: the quasi-Newton algorithm, the simplex algorithm, the Hooke and Jeeves pattern search method, and the Rosenbrock method of rotating coordinates [78].

The quasi-Newton method is based on the following: in every step of the iteration, a function is estimated in various points for the purpose of the estimation of first and second order derivatives. This information is subsequently used in order to follow the path heading toward the minimum of the loss function. The quasi-Newton method is the most effective of the discussed methods (i.e., it provides the lowest value of the loss function in the smallest number of iterations). The simplest procedure is based on the estimation of the loss function derivatives. In every iteration, the function is estimated in m + 1 points in an m-dimensional space of parameters. In a two-dimensional space, the points form a triangle that “moves” toward the bottom of the loss function, until this function reaches a minimum. This method is less sensitive to local extrema than the quasi-Newton algorithm because the triangle is made bigger or smaller in the case of need in subsequent steps. In case of an m-dimensional space, the name of the figure is simplex. The Hooke–Jeeves method is based on relocating a whole set of points in an m-dimensional space by the distance of a step that is constantly changed and adjusted to minimize the loss function. In the Rosenbrock method of rotating coordinates, the space of parameters is rotated and one axis is aligned to the ridge. All the other axes remain orthogonal to this axis. If the loss function is unimodal and has detectable ridges pointing toward the function minimum, then the method will head precisely toward the function minimum. The search algorithm may be interrupted earlier if there are several limitations (penalty functions) that intersect, which leads to the discontinuity of the ridges. Sometimes the selection of a combination of methods delivers the best results of the estimation. The simplex, Hooke and Jeeves, and Rosenbrock methods are generally less sensitive to local minimums, so one can apply these methods together with the quasi-Newton method. This is especially convenient when there is no certainty regarding the appropriate initial values for the estimation. In such a case, the first method may generate the initial parameter estimators that will then be used in subsequent quasi-Newton method iterations. A significant problem of the construction of a non-linear model is the selection of the appropriate analytical form of the relationship. There is no universal method allowing for the determination of the analytical form of the function in every situation.

On the basis of the existing theory explaining the mechanism of the analyzed phenomenon, the a priori knowledge about the analyzed phenomenon is used, constructing characteristic equations (difference equations or differential equations), the solution to which are appropriate function classes. In the case of a lack of theoretical solutions, empirical material is used. Graphs of the empirical dispersion of points (sets of points) are designated in the coordinate system and on the basis of the shape of those graphs, the adequate analytical function form is selected. Such an approach may be used in the case of a model only with one explanatory variable. Mixed method if the theory referring to the considered phenomenon indicates, for example, that the explained variable should have a saturation level, then it is necessary to adjust the curves that have a horizontal asymptote to empirical data.

Due to a lack of theoretical solutions, the second method of procedure was adopted in the modeling. The best approximation results (the lowest value of the loss function) were obtained for the segment nonlinear regression model in which, for each of the fractions of concrete, the analytical form of a perfect square trinomial was adopted.

The model of segment nonlinear regression may be recorded in the following way [79]:

(16) $$\begin{array}{l} \rho_{i}=\left(\alpha_{11}m_{i}^{2}+\alpha_{12}m_{i}+\alpha_{13}\right)\cdot I_{1}\left(\rho_{i}\leq \rho_{1}\right)+\left(\alpha_{21}m_{i}^{2}+\alpha_{22}m_{i}+\alpha_{23}\right)\cdot I_{2}\left(\rho_{1}<\rho_{i}\leq \rho_{2}\right)+\\ \;+\left(\alpha_{31}m_{i}^{2}+\alpha_{32}m_{i}+\alpha_{33}\right)\cdot I_{3}\left(\rho_{2}<\rho_{i}\leq \rho_{3}\right)+\xi_{i} \end{array}$$

where *ρ_i_* is the density of the mineral skeleton in the ith step of the analysis; *m_i_* is the total mass of the mineral skeleton in the ith step of the analysis; **α**_11_, *α*_12_, *α*_13_ are the parameters of the perfect square trinomial of the 1st fraction; *α*_21_, *α*_22_, *α*_23_ are the parameters of the perfect square trinomial of the 2nd fraction; and *α*_31_, *α*_32_, *α*_33_ are the parameters of the perfect square trinomial of the 3rd fraction.

*I_j_*(*p*) is the logic value of sentence p for the jth fraction:

(17) $$I_{j}\left(p\right)=\left\{ \begin{array}{l} 0-when\;the\;sentense\;p\;is\;false;\\ 1-when\;the\;sentence\;p\;is\;true. \end{array}\right.$$

where *ρ*_1_, *ρ*_2_, *ρ*_3_ are the threshold values which are the maximum densities of the mineral skeleton for each fraction and *ξ_i_* is the model’s disturbance term.

An element that requires explanation is the emergence of a disturbance term in the general record of the model. The disturbance term is a random variable that is expressed in the form of differences between the actual values of density and the model ones (theoretical). It is an immanent part of the model and may occur in the model due to three reasons.

First, due to the fact that in the process of analyses in laboratory conditions other factors (of negligible significance) affecting density (e.g., purity or the homogeneity of the specimen) have not been taken into account. Second, the wrong analytical form of the model was adopted, and third, the measurement of variables was performed with errors, (e.g., the reading of values from a scale and the inaccuracy of measurement devices).

In the process of estimation of the parameters of the segment nonlinear regression model, the statistical data that were used constituted arithmetic averages calculated for the fractions on the basis of the results of 10 research series. Such a procedure results in annulling a high number of cases of non-systematic errors of measurements of a random nature.

This information is subsequently used in order to follow the path heading toward the minimum of the loss function. The process of estimating parameters achieved convergence after 28 iterations (loss function minimum). The results of the estimation are juxtaposed in Table 14.

The following model was obtained:

(18) $$\begin{array}{c} {\hat{\;\rho }}_{i}=0.0000311\mathrm{mi}2+0.01184\mathrm{mi}+0.53866\cdot I_{1}\left(\rho_{i}\leq 1.676\right)+\\ +0.0000665\mathrm{mi}2+0.03568\mathrm{mi}-2.57847\cdot I_{2}1.676<\rho i\leq 2.196+\\ +0.0000241\mathrm{mi}2+0.01498\mathrm{mi}-0.09403\cdot I_{3}2.196<\rho i\leq 2.244 \end{array}$$

**Figure 6:**
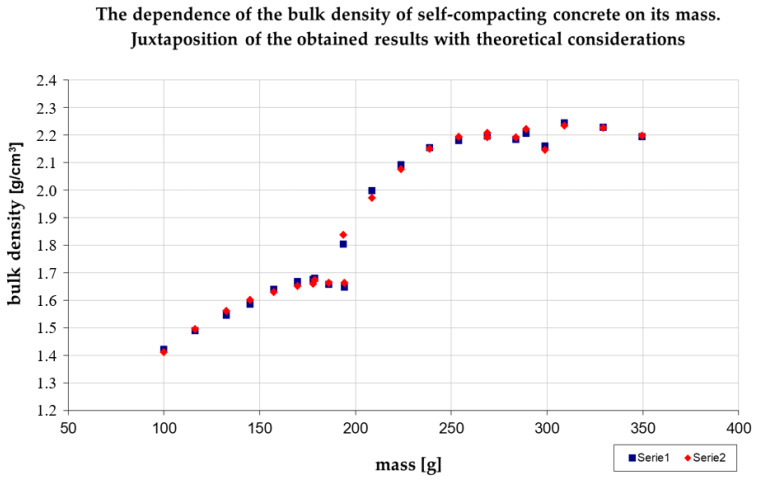
The dependence of the density of self-compacting concrete on its mass. Source: Own elaboration. Series 1—Theoretical values, Series 2—Empirical values determined in the analyses.

**Figure 7:**
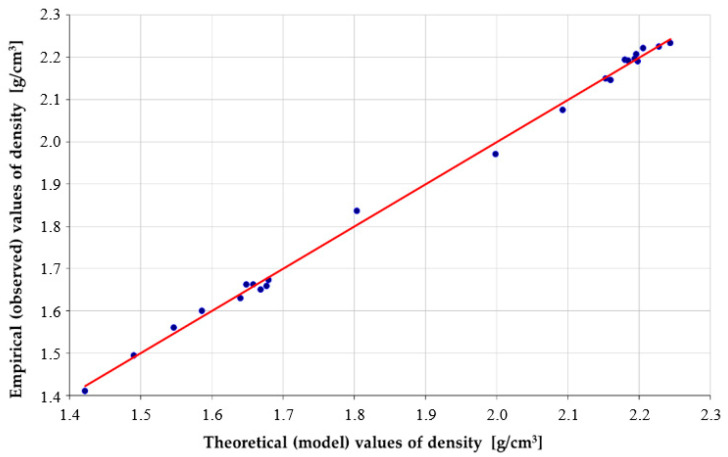
The empirical values compared to the theoretical ones obtained from the model (16).

The degree of explanation of the variance of the sought maximum density of the mineral mix by the model was very high and was equal to 99.75%. The theoretical values deviated from the empirical values on average only by 4.78 mg/cm^3^. The high degree of the model’s adjustment to the empirical data is also visible in Figure 6 and Figure 7.

The comparison between empirical values and the theoretical ones indicates very good properties of the phenomenon description by the obtained model. In the case of function dependences, the points are arranged along a line $\rho_{i}=a\hat{\rho_{i}}+b$, where *a* = 1 and *b* = 0.

The knowledge of the regression model of the mineral skeleton density in reference to its mass may be used in determining the tight structure of the concrete mineral skeleton. It is enough to—for each fraction—calculate the first order derivative in reference to mass and determine its zeros. Then, for the determined values of arguments (mass), the explained variable (density) will have the highest value. The density for the jth fraction may be calculated according to the following dependence:

(19) $${\hat{\rho }}_{ji}=\alpha_{j1}m_{i}^{2}+\alpha_{j2}m_{i}+\alpha_{j3}$$

Then, the first derivative has the following form:

(20) $${{\hat{\rho }}^{\prime }}_{ji}=2\alpha_{j1}m_{i}^{}+\alpha_{j2}$$

Thus, the density maximum is obtained for:

(21) $$m_{i}^{*}=\frac{-\alpha_{j2}}{2\alpha_{j1}}$$

Formula (21) allows for determining such proportions of concrete components for which maximum density is obtained in particular stages of the analysis.

### 3.8. Creating Trial Batches of the Designed Concrete Mix and the Verification of the Rheological Properties

Stage IV of designing self-compacting concrete consists of creating trial batches of the designed concrete mixes and in determining the self-compacting properties with the help of a three grade assessment based on the L-box, the V-funnel, and the Abrams inverted cone and in determining the mechanical properties after 90 days of concrete hardening.

**Figure 8:**
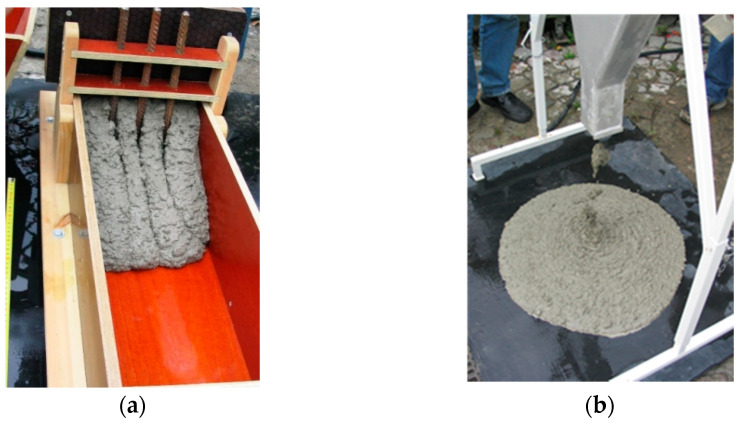
Analysis of self-compacting and flow dynamics performed using the L-box (**a**) and the V-funnel (**b**).

Analyses of the concrete mix were performed in accordance with the applicable standards and the results are juxtaposed in Table 15 and Figure 8.

Subsequently, after performing the analyses of the features of the fresh concrete mix, cubic specimens were created for the purpose of verifying the mechanical properties of these mixes after 90 days of hardening. The results obtained during the analyses are presented in Table 16.

The concrete mix prepared according to the SCC 4-G recipe met all the rheological properties required for the SCC mix. It demonstrated the flow of 72 cm and the time of outflow from the measurement funnel was equal to 6.5 s. The concrete mix prepared according to recipe no. 11 also met all the rheological properties required for the SCC mix and it demonstrated the flow of 70 cm and the time of outflow from the measurement funnel equal to 7.7 s.

### 3.9. Verification of the Bingham Model Using the Obtained Study Results

As a result of the studies carried out by the authors of the French method, Sedran and de Larrard [34], there is a possibility of estimating the basic parameters of the Bingham model (plastic viscosity and the yield strength) with the help of the study results obtained using the Abrams inverted cone. On the basis of my own studies (the measured flow diameter and the *t*_500_ time), the yield stress was determined according to Formula (22):

(22) $$\tau_{0}=\left(808-S_{1}\right)\cdot \frac{M\cdot g}{11740}$$

On the basis of my own studies (the measured flow diameter and the *t*_500_ time), the plastic viscosity was determined according to Formula (23):

(23) $$\mu =\frac{M\cdot g}{10000}\cdot \left(0.026\cdot S_{1}-2.39\right)\cdot t_{500}$$

The results of the verification are juxtaposed in Table 17.

**Figure 9:**
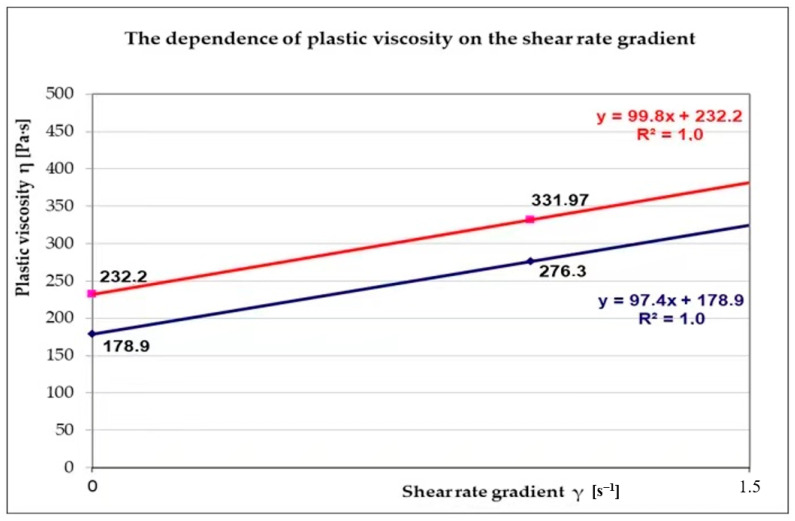
The dependence of plastic viscosity on the gradient of the shear rate of the concrete mix with the SCC 4-G and SCC 11-B recipe composition.

After analyzing the study results and estimating the basic parameters of the Bingham model (plastic viscosity and yield strength) with the help of the diameter and the dynamics of the flow obtained thanks to the Abrams inverted cone, a graphic verification of the Bingham model was performed (Figure 9).

### 3.10. Discussion

The article presents the functional method of designing self-compacting concrete. The proposed procedure takes into consideration such a selection of the mineral skeleton in terms of the volumetric saturation of the mineral skeleton, which prevents the blocking of aggregate grains, and the designed liquid phase demonstrated high structural viscosity and low yield stress. The performed experimental studies, the simulation of the elaborated mathematical model fully allowed for the verification of the theoretical assumptions that are the basis for the development of the method of designing self-compacting concrete.

The designed cement paste demonstrated lowered yield stress and structural viscosity, which prevented the segregation and sedimentation of the components of the mineral mix during flow and gravity compacting. On the basis of the conducted studies of the flow curves of specimens made from pastes (the continuous phase) in the rotary viscometer, clear deviations were noticed in reference to the Bingham liquid model declared in the literature. These deviations in reference to the model adopted in the literature were observed in case of two main phenomena. First, after exceeding the yield strength $\tau_{0}$, the vast majority of the paste solutions analysed in the rotary viscometer demonstrated pseudoplastic course (i.e., they were subject to shear thinning). Second, in the analyses in the closed cycle (i.e., with the increasing, and subsequently decreasing shear rate), the flow curves did not overlap and depending on the adopted recipe (the water–cement ratio, the amount of the inert filler as well as the amount and type of superplasticizer), they created a bigger or smaller viscosity hysteresis loop.

The size of the area of the viscosity hysteresis loop of the analyzed cement pastes achieved higher values for the CEM II cement. This cement included a 20% addition of blast-furnace slag and, therefore, is a binder of a greater thixotropic nature, whereas the selection of the BV 10 superplasticizer guaranteed certain thixotropic properties with the lowest dosage in reference to the mass of the cement (only 1%). This was possible thanks to applying a new generation of a very strongly liquidating polymer CP, composed of various kinds of oligomers that increase the viscosity of make-up water and limit the segregation of the components of the concrete mix mineral skeleton.

The selection of the CP1 polymer for the designed cement paste allowed for obtaining certain thixotropic properties with its minimum amount (the lowest dosage) in reference to the mass of the cement (only 1%). The decisive factors here were, above all, economical concerns and the informed avoidance of the problem of the repeated dosing of the superplasticizer at the construction site, which is typical for mixes with a high dose of the superplasticizer.

The designed mineral skeleton on the basis of coarse aggregate, fine aggregate (sand), and the inert filler demonstrated minimum free space. The tightly packed aggregate skeleton impacted the lowering of the demand for cement paste (i.e., water and cement), resulting in the increase of strength and resistance to the effect of freeze. The creation of a dispersive structure was achieved through the introduction of the dispersion rate *u,* which, by surrounding particular grains with a thin layer of the paste, allowed the concrete mix to free slide and flow without causing the phenomenon of coarse aggregate blocking. The grain size distribution curves of the designed mineral mixes were not located in the area of good grading applied during the design of the grading of ordinary concrete. The analysis of the graphic character of the grain size distribution curve allows for qualifying this type of mineral mix to the SMA (Stone Mastic Asphalt) asphalt mix, which is known and widely used worldwide and has a macadam-concrete structure with clear flattening (deficiency) of the sand fraction from 0.5 to 4.0 mm.

The experimental verification of the designed compositions of self-compacting concrete was performed with the use of the Abrams inverted cone, the L-box, and the V-Funnel, and the findings were that the best plastic properties as well as the best properties referring to strength and durability were demonstrated by the mix of the following composition: SCC 4-G and SCC 11-B.

## 4. Conclusions

The performed experimental studies, the simulation of the elaborated mathematical model, and the verification of the theoretical assumptions supported with the experimental part allow for the following conclusions:

1. A properly designed mineral skeleton demonstrating minimum free space causes a reduction of the demand for cement and results in an increase in compressive strength and an increase in the concrete durability.
2. The introduction of a dispersive structure through the increase in the amount of the *Z*_2_ paste allowed for the separation of the grains of the aggregate to the distance of layer b and resulted in free slide and flow of the mix, without the clear blocking of the aggregate.
3. The cement paste based on the CEM II BV cement and the water–cement ratio (w/c) = 0.5 with 1% content of CP1 demonstrated reduced yield stress and viscosity typical for self-compacting concretes.
4. On the basis of the analyses of the flow curves of the analyzed pastes, deviations were noticed in reference to the Bingham model declared in the literature, demonstrating pseudoplastic course.
5. The concrete mixes prepared according to the SCC 4-G and SCC 11-B recipe met all the rheological properties required for the SCC mix. They demonstrated flow from 70 to 72 cm and the time of outflow from the measurement funnel from 6.5 to 7.7 s.
6. The obtained results of the studies of hardened concrete met the designed concrete class C30/37 with much reserve and demonstrated resistance to the effect of freeze.

## Figures and Tables

**Table 1:** The stages of the functional method of designing self-compacting concrete (SCC).

Stage	Description	Aim of Stage
Stage 1	Determining the water–cement ratio (w/c), the type of cement and the dose of the superplasticizer with the help of the Southard viscometer and a rotary viscometer.	The designed paste should demonstrate high viscosity and a high value of the surface area of the hysteresis loop of viscosity
Stage 2	The selection of the grading of the mineral mix performed using the maximum bulk density method	Obtaining a mineral mix which demonstrates the minimum amount of free spaces
Stage 3	Calculating the specific surface area of the aggregate skeleton and adopting the thickness of aggregate grain coating in order to separate the tightly distributed grains at the distance of the membrane b	Determining the minimum amount of the paste which will allow for the flow of the concrete mix without the blocking of aggregate grains
Stage 4	Creating the trial batch, performing an analysis of the concrete mix and of hardened concrete	Verifying the properties of the SCC (L-box, V-funnel, the Abrams inverted cone, analyses of properties related to strength and durability)

**Table 2:** The specific surface area of the pebble aggregate with the following density of 2.65 kg/dm^3.^

F_i_ Fraction	Range	Specific Surface Area
	(mm)	(cm^2^/g)
F_1_	0/0.063	640
F_2_	0.063/0.125	320
F_3_	0.125/0.25	160
F_4_	0.25/0.5	80
F_5_	0.5/1	40
F_6_	1/2	20
F_7_	2/4	10
F_8_	4/8	5
F_9_	8/16	2.5
F_10_	16/31.5	1.25
F_11_	31.5/63	0.625

**Table 3:** The viscosity of the paste in the rotary viscometer.

	The Viscosity of the Paste in the Rotary Viscometer				
Cement/w/c	0.40	0.45	0.50	0.55	0.60
CEM I 32.5 R	42.52	37.80	33.07	28.35	23.62
CEM II/B-S 32.5	51.97	40.16	42.52	33.07	28.35

**Table 4:** The flow of the paste in the Southard viscometer.

	The Flow of the Paste in the Southard Viscometer (cm)				
Cement/w/c	0.40	0.45	0.50	0.55	0.60
CEM I 32.5 R	-	8	12	16	20
CEM II/B-S 32.5	-	11	14	17	22

**Table 5:** The viscosity of the paste in the rotary viscometer.

	The Viscosity of the Paste in the Rotary Viscometer			
Superplasticizer	0.50%	1.00%	1.50%	2.00%
CP1	293	408	398	255
CP2	248	305	315	158
CP3	273	236	142	210

**Table 6:** The viscosity of the paste in the Southard viscometer.

	The Viscosity of the Paste in the Southard Viscometer			
Superplasticizer	0.50%	1.00%	1.50%	2.00%
CP1	17	22	24	25
CP2	16	18	21	21
CP3	16	19	21	23

**Table 7:** The experimental selection of the grading.

	Gravel 8/16	Gravel 2/8	Sand 0/2	Limestone Dust	In Total	Volume	Density
	(g)	(g)	(g)	(g)	(g)	(cm^3^)	(g/cm^3^)
1	250	0			250	173	1.421
2	250	30			280	185	1.491
3	250	60			310	198	1.545
4	250	90			340	212	1.585
5	250	120			370	224	1.634
6	250	150			400	238	1.664
7	250	180			430	254	1.677
8	250	210			460	274	1.664
9	250	240			490	295	1.647
10	200	146	0		346	206	1.681
11	200	146	30		376	208	1.809
12	200	146	60		406	206	1.972
13	200	146	90		436	210	2.078
14	200	146	120		466	217	2.149
15	200	146	150		496	228	2.178
16	200	146	180		526	240	2.194
17	200	146	210		556	252	2.208
18	200	146	240		586	268	2.189
19	200	146	270		616	285	2.165
20	150	109.5	158	0	417.5	190	2.209
21	150	109.5	158	15	432.5	195	2.219
22	150	109.5	158	30	447.5	200.6	2.232
23	150	109.5	158	45	462.5	207	2.236
24	150	109.5	158	60	477.5	214	2.233
25	150	109.5	158	75	492.5	221.5	2.225
26	150	109.5	158	90	507.5	229	2.217

**Table 8:** The compositions of mineral mixes determined on the basis of maximum bulk density.

Type of Material	Stage I	Stage II	Stage III	Stage I	Stage II	Stage III
gravel 8/16 mm	57.74%	35.97%	32.41%			
gravel 2/8 mm	42.26%	26.24%	23.66%			
sand 0/2 mm		37.79%	34.21%		33.69%	30.29%
limestone dust			9.72%			10.11%
basalt 8/16 mm				62.50%	40.43%	36.36%
basalt 2/8 mm				37.50%	25.88%	23.24%
in total	100%	100%	100%	100%	100%	100%
bulk density, g/cm^3^	1.682	2.208	2.236	1.954	2.409	2.420

**Table 9:** Specific density in the Le Chatelier Flask.

Type of Material	Specific Density in the Le Chatelier Flask, g/cm^3^
gravel 8/16 mm	2.65
gravel 2/8 mm	2.65
sand 0/2 mm	2.64
limestone dust	2.66
basalt 8/16 mm	2.95
basalt 2/8 mm	2.95

**Table 10:** Analysis of the grading of natural aggregates.

Sieve (mm)	Gravel 8/16 mm	Gravel 2/8 mm	Sand 0/2 mm	Limestone Dust	Basalt 8/16 mm	Basalt 2/8 mm
16.000	0.0	0.0	0.0	0.0	0.8	0.0
8.000	71.0	2.9	0.0	0.0	96.3	4.1
4.000	21.1	39.0	0.0	0.0	2.9	67.2
2.000	6.2	41.0	0.0	0.0	0.0	26.9
1.000	1.7	11.0	9.7	0.0	0.0	1.8
0.500	0.0	6.1	21.0	0.0	0.0	0.0
0.250	0.0	0.0	39.0	0.0	0.0	0.0
0.125	0.0	0.0	25.0	2.0	0.0	0.0
0.000	0.0	0.0	5.3	98.0	0.0	0.0
in total	100.0	100.0	100.0	100.0	100.0	100.0

**Table 11:** Determining the demand for *Z*_2_ paste.

Fraction	Fraction Specific Surface Area *f_i_*	Grain Size Distribution Curve	The Calculated Specific Surface Area *P_w_*	Coating Radius *b*	*P_w_·b*	Paste Density	Demand for *Z*_2_ Paste
(mm)	(cm^2^/g)	(%)	(cm^2^/g)	(cm)	(cm^3^/g)	(g/cm^3^)	(g_z_/g_k_)
31.5–16.0	0.625	0.0000	0.00	0.005	0.0000	1.81	0.0000
16.0–31.5	1.25	0.0000	0.00	0.005	0.0000	1.81	0.0000
8.0–16.0	2.5	0.2370	0.59	0.005	0.0030	1.81	0.0054
4.0–8.0	5	0.1607	0.80	0.005	0.0040	1.81	0.0073
2.0–4.0	10	0.1171	1.17	0.005	0.0059	1.81	0.0106
1.0–2.0	20	0.0647	1.29	0.00006	0.0008	1.81	0.0014
0.5–1.0	40	0.0863	3.45	0.0006	0.0021	1.81	0.0037
0.25–0.5	80	0.1334	10.67	0.0006	0.0064	1.81	0.0116
0.125–0.25	160	0.0875	13.99	0.0006	0.0084	1.81	0.0152
0.063–0.125	320	0.0378	12.10	0.0006	0.0073	1.81	0.0131
0–0.063	640	0.0756	48.39	0.0006	0.0290	1.81	0.0526
		1.0000	92.5				0.1217

**Table 12:** The designed composition of self-compacting concretes.

No. of Recipe.	Cement	Limestone Dust	Water	CP1	0/2	2/8	8/16
	(kg/m^3^)	(kg/m^3^)	(kg/m^3^)	(kg/m^3^)	(kg/m^3^)	(kg/m^3^)	(kg/m^3^)
SCC 4-G	318	189	159	3.2	666	460	631
SCC1 11-B	300	213	150	4.5	639	490	767

**Table 13:** Juxtaposition of recipe compositions.

**Gravel**	**Cement**	**Limestone Dust**	**Water**	**CP1**	**0/2 Sand**	**2/8 Gravel**	**8/16 Gravel**
	****(kg/m^3^)****	**(kg/m^3^)**	**(kg/m^3^)**	**(kg/m^3^)**	**(kg/m^3^)**	**(kg/m^3^)**	**(kg/m^3^)**
SCC 1-G	318	0	159	3.2	737	510	699
SCC 2-G	318	67	159	3.2	712	492	674
SCC 3-G	318	130	159	3.2	688	476	652
SCC 4-G	318	189	159	3.2	666	460	631
SCC 5-G	318	244	159	3.2	645	446	611
SCC 6-G	318	296	159	3.2	625	433	593
SCC 7-G	318	345	159	3.2	607	420	575
**Basalt**	**Cement**	**Limestone Dust**	**Water**	**CP1**	**0/2 Sand**	**2/8 Gravel**	**8/16 Gravel**
	**(kg/m^3^)**	**(kg/m^3^)**	**(kg/m^3^)**	**(kg/m^3^)**	**(kg/m^3^)**	**(kg/m^3^)**	**(kg/m^3^)**
SCC 8-B	300	0	151	4.5	715	548	858
SCC 9-B	300	76	151	4.5	688	528	825
SCC 10-B	300	147	150	4.5	663	508	795
SCC 11-B	300	213	150	4.5	639	490	767
SCC 12-B	300	275	150	4.5	617	474	741
SCC 13-B	300	332	150	4.5	597	458	717
SCC 14-B	300	386	150	4.5	578	443	694

**Table 14:** The results of estimating the structural parameters of the segment nonlinear regression model.

$L=\sum_{i=1}^{n}{\left(\rho_{i}-{\hat{\rho }}_{i}\right)}^{2}$ The Summing-Up of the Segment Nonlinear Regression of Concrete Density: *ρ_i_*; Loss:									
Final Loss = 0.004775336; R = 0.9987; Explained Variance: 99.75%									
Assessments of Parameters:									
Threshold Value:	${\hat{\alpha }}_{11}$	${\hat{\alpha }}_{12}$	${\hat{\alpha }}_{13}$	${\hat{\alpha }}_{21}$	${\hat{\alpha }}_{22}$	${\hat{\alpha }}_{23}$	${\hat{\alpha }}_{31}$	${\hat{\alpha }}_{32}$	${\hat{\alpha }}_{33}$
1.676	−3.12 × 10^−5^	0.01184	0.53866						
2.196				−6.65 × 10^−5^	0.03568	−2.57847			
2.244							−2.41 × 10^−5^	0.01498	−0.09403

**Table 15:** Juxtaposition of the results of the analyses of the rheological features of concrete mixes.

	T_50_	(R_1_+R_2_)/2	T _funnel_	(R_3_+R_4_)/2	T_20_	T_40_	H_2_/H_1_
	(s)	(cm)	(s)	(cm)	(s)	(s)	
SCC 1-G	5.2	52	13.5	65	1.6	4.5	0.55
SCC 2-G	4.0	61	8.8	73	1.0	4.4	0.77
SCC 3-G	3.1	64	7.0	80	1.5	3.3	0.93
SCC 4-G	2.5	72	6.5	85	2.0	4.2	0.98
SCC 5-G	2.0	74	8.0	87	2.5	5.1	0.99
SCC 6-G	3.6	63	10.5	77	2.8	6.0	0.85
SCC 7-G	4.4	59	14.5	79	4.5	8.0	0.79
SCC 8-B	6.2	42	-	-	2.9	-	-
SCC 9-B	5.1	54	12.1	70	3.3	9.1	0.45
SCC 10-B	3.7	61	9.3	77	2.5	5.8	0.62
SCC 11-B	2.5	70	7.7	84	1.8	4.6	0.88
SCC 12-B	2.9	71	6.5	88	1.9	4.0	0.90
SCC 13-B	4.0	62	8.9	80	2.6	5.2	0.83
SCC 14-B	3.3	64	11.3	73	3.3	8.0	0.74

**Table 16:** Juxtaposition of the results of analyses of the rheological features of concrete mixes.

	f_c 7_	f_c 28_	f_c 90_	N	ΔM	ΔR
	(MPa)	(MPa)	(MPa)	(%)	(%)	(%)
SCC 1-G	28.5	40.1	49.3	4.7	0.9	29.9
SCC 2-G	32.1	44.2	54.2	4.5	0.55	23.1
SCC 3-G	29.7	46.2	43.7	4.1	0.42	18.4
SCC 4-G	31.3	44.9	56.2	3.7	0.61	11.5
SCC 5-G	30.3	45.1	55.3	3.5	0.42	9.8
SCC 6-G	25.1	41.0	49.6	4.0	0.87	19.2
SCC 7-G	20.4	38.3	50.9	3.9	0.72	22.1
SCC 8-B	36.5	51.2	58.3	4.3	0.11	30.3
SCC 9-B	32.4	48.8	61.1	3.9	0.20	20.6
SCC 10-B	33.9	52.1	61.9	3.8	0.17	14.1
SCC 11-B	31.6	54.0	64.4	3.5	0.09	17.2
SCC 12-B	32.2	57.9	66.0	3.7	0.10	9.8
SCC 13-B	30.8	55.4	64.5	3.6	0.06	16.0
SCC 14-B	28.9	50.3	62.4	4.1	0.24	24.0

**Table 17:** Determining the yield stress and plastic viscosity on the basis of analyses performed using the Abrams inverted cone.

	Flow	Concrete Density	Earth’s Gravity	Yield Stress	Flow Time	Plastic Viscosity
	(mm)	(kg/m^3^)	(m/s^2^)	(Pa)	(s)	(Pas)
SCC 1-G	520	2431.8	9.81	585.2	5.2	138.03
SCC 2-G	610	2432.1	9.81	402.3	4.0	128.55
SCC 3-G	640	2432.4	9.81	341.5	3.1	105.41
SCC 4-G	720	2432.7	9.81	178.9	2.5	97.40
SCC 5-G	740	2433.0	9.81	138.2	2.0	80.44
SCC 6-G	630	2433.2	9.81	361.8	3.6	120.22
SCC 7-G	590	2433.5	9.81	443.2	4.4	136.01
SCC 8-B	420	2586.2	9.81	838.5	6.2	134.16
SCC 9-B	540	2581.5	9.81	578.1	5.1	150.45
SCC 10-B	610	2577.1	9.81	426.3	3.7	125.99
SCC 11-B	700	2573.1	9.81	232.2	2.5	99.77
SCC 12-B	710	2569.3	9.81	210.4	2.9	117.43
SCC 13-B	620	2565.7	9.81	403.1	4.0	138.23
SCC 14-B	640	2562.4	9.81	359.7	3.3	118.22

## Data Availability

The data presented in this study are available on request from the corresponding author.
